# Transcriptomic and metabolomic insights into the antimicrobial mechanisms of *Murraya paniculata* (L.) Jack leaf extract

**DOI:** 10.3389/fpls.2025.1717793

**Published:** 2025-12-02

**Authors:** Qing Ma, Lin Zhang, Azhen Nie, Yini Shi, Zhongqiu Liu, Rongrong Zhang, Zhongke Sun

**Affiliations:** 1 International Institute for Translational Chinese Medicine, School of Pharmaceutical Sciences, Guangzhou University of Chinese Medicine, Guangzhou, China; 2 China Resources Sanjiu Medical and Pharmaceutical Co., Ltd, Shenzhen, China; 3 Guangzhou Medical University, State Key Laboratory of Respiratory Disease, The First Affiliated Hospital of Guangzhou Medical University, Guangzhou, China; 4 School of Biological Engineering, Henan University of Technology, Zhengzhou, China

**Keywords:** *Murraya paniculata*, antimicrobial activity, transcriptomics, metabolomics, antibiotics

## Abstract

The small tropical evergreen shrub, *Murraya paniculata* (L.) Jack (*M. paniculata*) exhibits inhibitory effects against a range of pathogens. However, the molecular basis for this antimicrobial activity is largely unknown. This study investigated how *M. paniculata* inhibits bacterial growth. Five different extracts showed variable antimicrobial potentials against four bacterial pathogens. The acetone extract of *M. paniculata* leaf (AEML) inhibited the growth of all pathogens, with minimum inhibitory concentration (MIC) values ranging from 200 to 400 μg/mL. Further assays on pathogenic *Escherichia coli* showed dose-dependent effects involving disruption of the cell wall and membrane, as indicated by increased secretion of intracellular components and propidium iodide staining. Transcriptomic analysis demonstrated that AEML regulated bacterial gene expression (357 genes upregulated and 280 downregulated), with most differentially expressed genes enriched in oxidative phosphorylation and the citrate cycle. In particular, downregulated metabolisms of thiamine and biotin metabolism—cofactors essential for energy metabolism—was downregulated. Finally, untargeted metabolomic analysis identified more than 1,000 metabolites in AEML by LC-MS/MS, including phenols and flavonoids contributing to the antimicrobial effect. Notably, more than 30 different antibiotics were detected. Taken together, *M. paniculata* produces versatile antimicrobial agents that exert profound effects on bacterial physiology. These findings provide novel molecular insights into the antimicrobial effects of *M. paniculata.*

## Graphical Abstract

**Figure:**
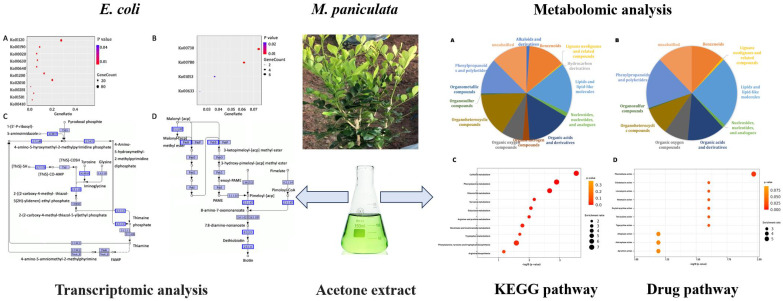

## Highlights

- The antimicrobial effect of *Murraya paniculata* on *E. coli* is partially due to disruption of the cell wall and membrane.
- The acetone extract of *M. paniculata* leaf downregulated metabolisms of thiamine and biotin metabolism in *E. coli.*
- More than 1,000 metabolites were identified from the acetone extract of *M. paniculata* leaf.
- The acetone extract of *M. paniculata* leaf contained more than 30 antibiotics.

## 1 Introduction

*Murraya paniculata* (L.) Jack (*M. paniculata*) is a shrub species of shrub that is widely cultivated for its fragrant and attractive bright yellow flowers (www.nparks.gov.sg). Besides its ornamental value, the plant has been used for centuries as an herbal medicine to treat a range of ailments, including abdominal pain, diarrhea, stomach ache, headache, edema, thrombosis, and blood stasis. In traditional Chinese medicine, *M. paniculata* has been used to treat conditions such as fever, inflammation, and digestive disorders. In Ayurvedic medicine, the plant has been used to relieve symptoms of colds, coughs, and sore throats. Other reported uses include the treatment of skin diseases, wounds, and insect bites (Qi et al., 2024). In recent years, scientific research has begun to validate these traditional uses, revealing the plant’s potential as a source of bioactive compounds with various pharmacological effects (Joshi and Gohil, 2023). *M. paniculata* contains a variety of bioactive compounds, including alkaloids, flavonoids, terpenoids, and essential oils (Dosoky et al., 2016; Liang et al., 2021; Rehman et al., 2023). These compounds are thought to be responsible for the plant’s medicinal properties, such as antimicrobial, antiviral, and anti-inflammatory activities (Yohanes et al., 2023).

Infectious diseases remain a major global health concern, causing millions of deaths each year (Baker et al., 2022). Antibiotics are powerful tools for controlling infectious diseases; however, they can have negative side effects, such as triggering allergic reactions or disrupting the normal balance of bacteria in the body (Blumenthal et al., 2019; Ramirez et al., 2020). Moreover, the widespread use of antibiotics in human and veterinary medicine accelerates the development of antibiotic resistance (Larsson and Flach, 2022). Therefore, the evaluation of new antimicrobial agents is of significant importance for developing new therapeutic strategies (Baran et al., 2023). Many natural constituents, including plant extracts, continue to be used in modern medicine to treat a wide variety of infectious diseases (Jiang et al., 2022). In fact, the antimicrobial activity of *M. paniculata* was reported many years ago (Gautam et al., 2012; Rodríguez et al., 2012). The essential oil and other extracts of *M. paniculata* have shown activity against a range of microorganisms, including bacteria, fungi, and viruses.

To date, different extracts of *M. paniculata* have exhibited a broad-spectrum of antimicrobial activities against various pathogenic strains. For example, it was found that the coumarin compounds murrangatin and murrangatin diacetate, isolated from the ethyl acetate extract of *M. paniculata* leaves, demonstrated stronger antibacterial potency against *Porphyromonas gingivalis* than the crude extract (Rodanant et al., 2015). The ethanol extract of *M. paniculata* leaves inhibited the growth of *Staphylococcus aureus* (Panda et al., 2020). Volatile oils inhibited the growth of *Aspergillus niger* (*A. niger*), *A. fumigatus*, *Fusarium solani*, *Sclerotinia sclerotiorum, Mycobacterium kansasii* (*My. kansasii*), and *My. tuberculosis*, demonstrating broad antifungal and antimycobacterial activities (Selestino Neta et al., 2017; Silva et al., 2019). Volatile oils also showed promising antibacterial activity against *My. smegmatis*, *Pseudomonas aeruginosa*, and *Klebsiella pneumoniae* (Rodríguez et al., 2012; Saikia et al., 2021). However, details on the mechanisms underlying the antimicrobial effect of *M. paniculata* remain scarce.

*M. paniculata* is officially recognized as a source of Murrayae Folium et Cacumen (MFC) in the Chinese Pharmacopoeia. The dried leaves and twigs of *M. paniculata* are the major tissues used for the production of several Chinese patent medicines, such as Sanjiu Weitai Granule, which holds approximately three billion market shares. Although liquid chromatography coupled with mass spectrometry (LC-MS/MS) has recently been applied to compare M. *paniculata* and *M. exotica*, only 200 compounds were identified (Liang et al., 2024). Moreover, there is no available systematic analysis of the nonvolatile metabolomes of *M. paniculata*, and the antimicrobial substances in its leaves remain largely unknown. Currently, transcriptomics is widely used to evaluate gene expression under different conditions, while untargeted metabolomics is applied for the discovery of new metabolites (Kumari et al., 2024; Wang et al., 2025).

Therefore, we tested the antimicrobial activities of five different extracts against four pathogenic strains. The secretion of nucleic acids and proteins into the culture supernatant of *Escherichia coli* (*E. coli*) was assessed, and bacterial cells were also stained after treatment. To uncover the molecular mechanism underlying the antimicrobial effect of *M. paniculata*, we performed transcriptomic analysis was performed to probe the genetic response of *E. coli* in the presence of *M. paniculata* leaf extract. We further conducted untargeted metabolomic analysis of *M. paniculata* leaves to clarify the biochemical basis of the antimicrobial effect. Several potential antimicrobial substances were thoroughly examined, including phenols, flavonoids, organic acids, antibiotics, and other antimicrobial agents. This study aims to provide a phytochemical basis and novel molecular insights into the antimicrobial mechanisms of *M. paniculata.*

## 2 Materials and methods

### 2.1 Chemicals, media, and microorganisms

Ammonium acetate (NH_4_AC) was ordered from Sigma (Cat. 73594, Sigma-Aldrich, Shanghai, China). Acetonitrile was ordered from Merk (Cat. 1499230-935). Ammonium hydroxide (NH_4_OH), methanol (Cat. M813902), ethanol (Cat. M809064), and hexane (Cat. H810749) were purchased from Macklin (Macklin Inc., Shanghai, China). All microbial strains were glycerol stocks purchased from the Guangdong Institute of Microbiology Culture Center (GIMCC, Guangzhou, China). These strains were *Staphylococcus aureus* ATCC 6538 (*S. aureus*), *Salmonella enterica* subsp. enterica serotype Typhimurium ATCC 14028 (*Sa. typhimurium*), *Escherichia coli* ETEC GDMCC NO.1.4025 (*E. coli*), and *Streptococcus porcinus* GDMCC NO.1.1044 (*St. porcinus*). *St. porcinus* was grown in TSA (Cat. HB0177) or TSB (Cat. HB4114) supplemented with 5% off-fiber sheep blood (Cat. 1001339-1). All other strains were grown in LB broth or agar media at 37°C. All media and off-fiber sheep blood were ordered from a company (Hopebio Com. Ltd**. **Qingdao, China).

### 2.2 Preparation of plant extracts with different solvents

The leaves of *M. paniculata* growing in the wild were collected in March 2023 from the Yunfu planting base in Guangzhou, China. The plant was identified by Zhengzhou Han, Director of the Medical Plant Resources Department, Shenzhen Traditional Chinese Medicine Manufacturing Innovation Center Co., Ltd., Shenzhen, China. A voucher specimen (number HAUT-999-02) was deposited in the School of Biological Engineering, Henan University of Technology, Zhengzhou, China. Healthy and intact leaves were washed under tap water, shade dried, and ground into fine powder in liquid nitrogen. With a solid-to-solvent ratio of 20, an amount of 2.0 g leaf powder was mixed with 40 mL solvent, including hexane, acetone, ethanol, methanol, and water. In 50 mL plastic tubes, the samples were agitated for 12 h under sealed agitation at 150 rpm and 30°C under sealed conditions. Samples were then transferred into a Soxhlet extractor and boiled for approximately 8 h for two cycles (4 h per cycle) with three technical repeats. After rotary evaporation, the samples were resuspended in a final volume of 10 mL in the corresponding solvent and separated using filter papers. Finally, the filtered solutions were freeze dried under reduced pressure in a lyophilizer (Ecomini-60, Nanjing Jinshi Instrument Equipment, Nanjing, China). The freeze-dried extract was stored in sealed tubes packaged with a black bag at 4°C until future use.

### 2.3 Antimicrobial potential evaluation and MIC assays using pure culture

The antibacterial activity of the extracts was evaluated using the agar dilution method, as recommended by the National Committee for Clinical Laboratory Standards (CLSI M07, 12^th^ Edition), with four bacterial strains. Except for *St. porcinus*, which was propagated in TSB supplemented with 5% sheep blood, the other three pathogens were suspended in LB broth. Growth was maintained in sterile glass tubes by agitation at 150 rpm for 24 h, at 37°C. The cultures were then diluted to 1 × 10^6^ CFU/mL in phosphate-buffered saline (PBS). A 1 mL aliquot of the suspension was mixed with 20 mL of different media in plates. After solidification of the media, holes were punched under sterile conditions, and 100 μL of each extract at 20 mg/mL was loaded. Plates were incubated for 24 h at 37°C, and inhibition zones were measured. The MIC of *M. paniculata* leaf extract was defined as the lowest concentration at which.

### 2.4 Test the impacts of AEML on *E. coli* growth and cell permeability

The acetone extract of *M. paniculata* leaf (AEML, 20 mg/mL) was used to test the dose-dependent growth inhibition effect. Briefly, overnight-cultivated *E. coli* was inoculated into 5 mL LB medium supplemented with different volumes of *M. paniculata* leaf extract, to achieve final concentrations ranging from 0 to 400 μg/mL. With an initial OD_600_ = 0.01, the incubation was carried out by shaking at 150 rpm at 37°C for 12 h. An aliquot of 200 μL was taken every 2 h and diluted with 800 μL H_2_O for OD_600_ measurement using a Multimode Reader Spark^®^ (Tecan, Groedig, Austria).

Cell permeability in samples treated with 0 or 200 μg/mL AEML was evaluated using commercial propidium iodide (PI) staining solution (Yeasen Biotechnology Co., Ltd., Shanghai, China). Stained samples were imaged with an automated digital inverted microscope (Thermo Fisher EVOS M7000 Imaging System, USA) to capture fluorescence signals. In addition, total contents of protein and nucleic acid contents in the cell-free supernatants were assayed by measuring Abs. 280 nm and Abs. 260 nm using the Tecan multimode reader. Cell-free supernatants were prepared by centrifugation (5,000 g, 2 min) of *E. coli* cultures in the absence or presence of AEML.

### 2.5 Transcriptomic study of *E. coli* after treatment with AEML

The pathogenic strain *E. coli* ETEC GDMCC NO.1.4025 was propagated in 5 mL LB broth supplemented with or without 200 μg/mL AEML. Bacterial growth was carried out under aerobic conditions at 37°C for 12 h. Cells were harvested from triplicate cultures by centrifugation at 10,000 g for 2 min at 4°C for RNA isolation. TRIzol^®^ Reagent (Thermo Fisher) was used for total RNA extraction. RNA concentration was measured using a Nanodrop 2000, purity was verified by agarose gel electrophoresis and the RNA Integrity Number (RIN) was 8.3 jar (Origingene, v1.0).

### 2.6 Bioinformatic analysis of transcriptomic data

To ensure the accuracy of subsequent bioinformatics analysis, the raw sequencing data were first subjected to quality filtering to generate high-quality controlled data (clean data. Specific steps included: (1) removal of adapter sequences; (2) removal of bases at the 5′ end that did not contain AGCT before cleavage; (3) trimming of low-quality bases (sequencing quality score < Q20) from the ends; (4) removal of reads with an N content ratio ≥ 10%; and (5) discarding of fragments shorter than 25 bp after adapter removal and quality trimming.

All clean reads were annotated by BlastX alignment in different databases, including NR, STRING, COG, and KEGG, using the Diamond v0.9.19.120 with an E-value < 1e−5. For differential expression analysis, a gene-level raw read count matrix was constructed using edgeR. All analytical steps followed the standard workflow of edgeR v3.24 (http://www.bioconductor.org/packages/release/bioc/html/edgeR.html), including data filtering, normalization (using the TMM method), and fitting of generalized linear models (GLMs) with a default threshold of a false discovery rate p < 0.05 and |log2FC| > 1. The transcripts per million reads (TPM) expression matrix was used for downstream visualization analyses, such as principal component analysis (PCA) plots and heatmaps, to visually illustrate the overall expression similarity among samples and gene expression patterns. GO and KEGG pathway enrichment analyses of DEGs were conducted by Origingene using self-developed software.

### 2.7 Untargeted metabolomic analysis of AEML by LC-MS/MS

A total of 60 healthy and intact leaves were randomly collected from different sites of the Yunfu planting base. Every ten fresh leaves were quickly frozen in liquid nitrogen immediately and ground into fine powder with a mortar and pestle. A volume of 1 mL acetone was added to 100 mg plant powder for metabolite extraction. The mixture was agitated for 30 min and then centrifuged for 20 min (14,000 g, 4°C). The supernatant was dried in a vacuum centrifuge.

For LC-MS/MS analysis, the samples were re-dissolved in 100 μL acetonitrile/water (1:1, v/v) solvent and centrifuged at 14,000 g at 4°C for 15 min; the supernatant was then injected. To monitor the stability and repeatability of instrument analysis, quality control (QC) samples were prepared by pooling 10 μL of each sample and analyzed together with the other samples. The QC samples were inserted regularly and analyzed in every 5 samples.

Metabolomic analysis was performed using a UHPLC system (1290 Infinity LC, Agilent Technologies) coupled to a quadrupole time-of-flight mass spectrometer (AB Sciex TripleTOF 6600). The parameters were set as follows: collision energy (CE), 35 V with ± 15 eV; declustering potential (DP), 60 V (+) and −60 V (−); exclusion of isotopes within 4 Da; and 10 candidate ions monitored per cycle.

To validate the synthesis of antimicrobial substances, four antibiotics—ampicillin, norfloxacin, doxorubicin, and tigecycline—were detected by ELISA using corresponding commercial kits (Shanghai Enzyme-linked Biotech. Co., Ltd., Shanghai, China).

### 2.8 Processing of MS data and compound identification

The raw MS data were converted to MzXML files using ProteoWizard MSConvert before import into the freely available XCMS software. Quality control was performed by comparing the total ion chromatogram, principal component analysis, Pearson correlation, Hotelling’s T² test, multivariate control charts, and relative standard deviation of QC samples. To summarize metabolic profiles of all samples, total ion chromatograms (TICs) and extracted ion chromatograms (EICs or XICs) of QC samples were exported.

Each chromatographic peak area was determined. For peak picking, the following parameters were used: centWave m/z = 10 ppm, peakwidth = c (10, 60), prefilter = c (10, 100). For peak grouping, bw = 5, mzwid = 0.025, and minfrac = 0.5 were applied. The CAMERA (Collection of Algorithms of Metabolite Profile Annotation) package was used for annotation of isotopes and adducts. In the extracted ion features, only the variables with more than 50% of the nonzero measurement values in at least one group were retained. Compound identification of metabolites was performed by comparing the accurate m/z value (<10 ppm) and MS/MS spectra with an in-house database established using available authentic standards. Identification results were strictly checked and confirmed by manual secondary inspection. The identification level was above level 2. Level 2 is defined as matched to literature data or databases with diagnostic evidence and at least two orthogonal pieces of information, including evidence excluding all other candidates. Metabolites were compared with free online databases KEGG (http://www.genome.jp/kegg/) and HMDB (http://www.hmdb.ca/), and the corresponding KEGG pathways were extracted. Enrichment analysis was performed using MetaboAnalyst with different parameter settings (www.metaboanalyst.ca).

### 2.9 Statistical analysis

Experimental data were analyzed using the Origin 8.5 software. Measurement data were expressed as mean ± SD. Comparisons were performed using ANOVA in the IBM SPSS Statistics 25.0 software when necessary. The value of *p* ≤ 0.05 was set as the threshold for statistical significance.

## 3 Results and discussion

### 3.1 Antimicrobial potentials of different extracts of *M. paniculata* leaf

*M. paniculata* extract has been traditionally used as an antimicrobial medication and is believed to have significant antimicrobial activity. The inhibition effects of the ethanolic extract and hydroalcoholic extracts of *M. paniculata* leaves have been tested on several human pathogenic bacteria, including *E. coli, K. pneumoniae, E. faecalis, P. aeruginosa, Shigella flexneri, Shigella sonnei, Salmonella typhimurium*, and *Staphylococcus aureus.* However, only mild to moderate antimicrobial activity was demonstrated, and the methanol extract showed the highest antibacterial activity of 9–14 mm inhibition zones among other extracts at a concentration of 200 mg/mL (Gautam et al., 2012). At a much lower concentration (20 mg/mL), we observed different degrees of inhibition by different extracts (**Supplementary Table S1**). The AEML showed obvious antibacterial activity against all tested Gram-positive and Gram-negative bacteria. In contrast, the hexane extract showed no antibacterial activity, and the water extract inhibited only inhibits *Salmonella typhi*, consistent with a previous report showing that showed the leaf essential oil obtained by hydrodistillation exhibited no antibacterial activity (Dosoky et al., 2016). Although it was demonstrated that the essential oil of *M. paniculata* leaf was reported to be a promising antimicrobial agent due to its promising antibacterial activity against *My. smegmatis* and *P. aeruginosa* (MIC = 4 µg/mL), our determination of MIC results validate the agar diffusion observations: a MIC of 200 μg/mL was found for *S. aureus*, and higher MICs (400 μg/mL) were observed for the other three strains (Saikia et al., 2021).

**Figure 1:**
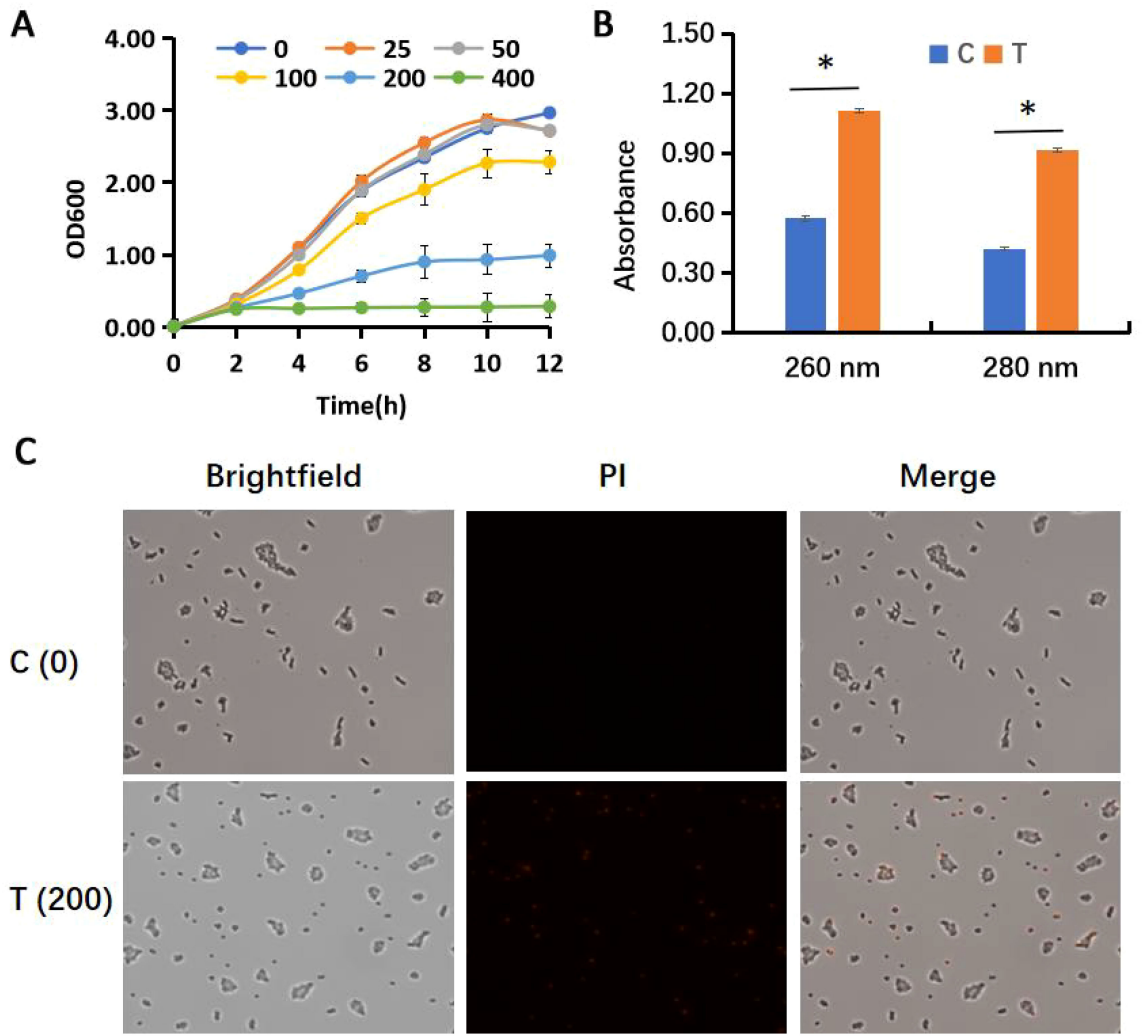
Antimicrobial effect of the acetone extract of *M. paniculata* leaf on *E. coli*: **(A)** the growth curves of *E. coli* in the presence of different concentrations (0–400 μg/mL) of leaf extract; **(B)** contents of nucleic acids and proteins in the supernatant after treatment with 200 μg/mL acetone extract of *M. paniculata* leaf; **(C)** PI staining of *E. coli* cells. In panel **(B, C)** C, control; T, treatment; PI, propidium iodide; *significantly different.

Due to the widespread presence of E. coli and its relevance to public infections, we further investigated the antimicrobial activity of AEML on this strain. Growth inhibition experiments indicated that AEML had little impact when the concentration was below 50 μg/mL, whereas an obvious inhibitory effect occurred when the concentration exceeded 100 μg/mL (**Figure 1A**). Notably, E. coli growth was completely inhibited when 400 μg/mL AEML was supplemented.

A common reason for the antibacterial effects is disruption of the bacterial cell wall and membrane disruption (Shan et al., 2020). Damage to these structures leads to leakage of intracellular components, such as nucleic acids and proteins (Zhou et al., 2022; Kawai et al., 2023). As shown in **Figure 1B**, the contents of nucleic acids and proteins are nearly doubled in the supernatant when 200 μg/mL AEML was added, suggesting leakage of these intracellular substances. Consistent with this, PI staining of E. coli cells directly demonstrated disruption of the cell wall and membrane, as red-fluorescent PI enters cells and binds genomic DNA (**Figure 1C**). These data clearly show that AEML has a direct impact on E. coli cell wall and membrane integrity.

### 3.2 Impacts of AEML on *E. coli* transcriptome

To obtain an overview of the genetic response of *E. coli* to AEML, we performed a comparative transcriptomic analysis. Statistics of RNA-seq statistics showed that as much as 36.419 Gb clean data were obtained, with an average of 6.07 Gb for each sample and Q30 > 97.8% (**Supplementary Table S2**). For each condition, three biological replicates were used, and the unique mapping rates ranged between 81.02% and 82.65%. Annotation of transcripts yielded a variable number of genes across different databases, ranging from 3,211 in KEGG to 5,010 in NR, with 2,145 genes shared across all databases (**Supplementary Table S3**).

**Figure 2:**
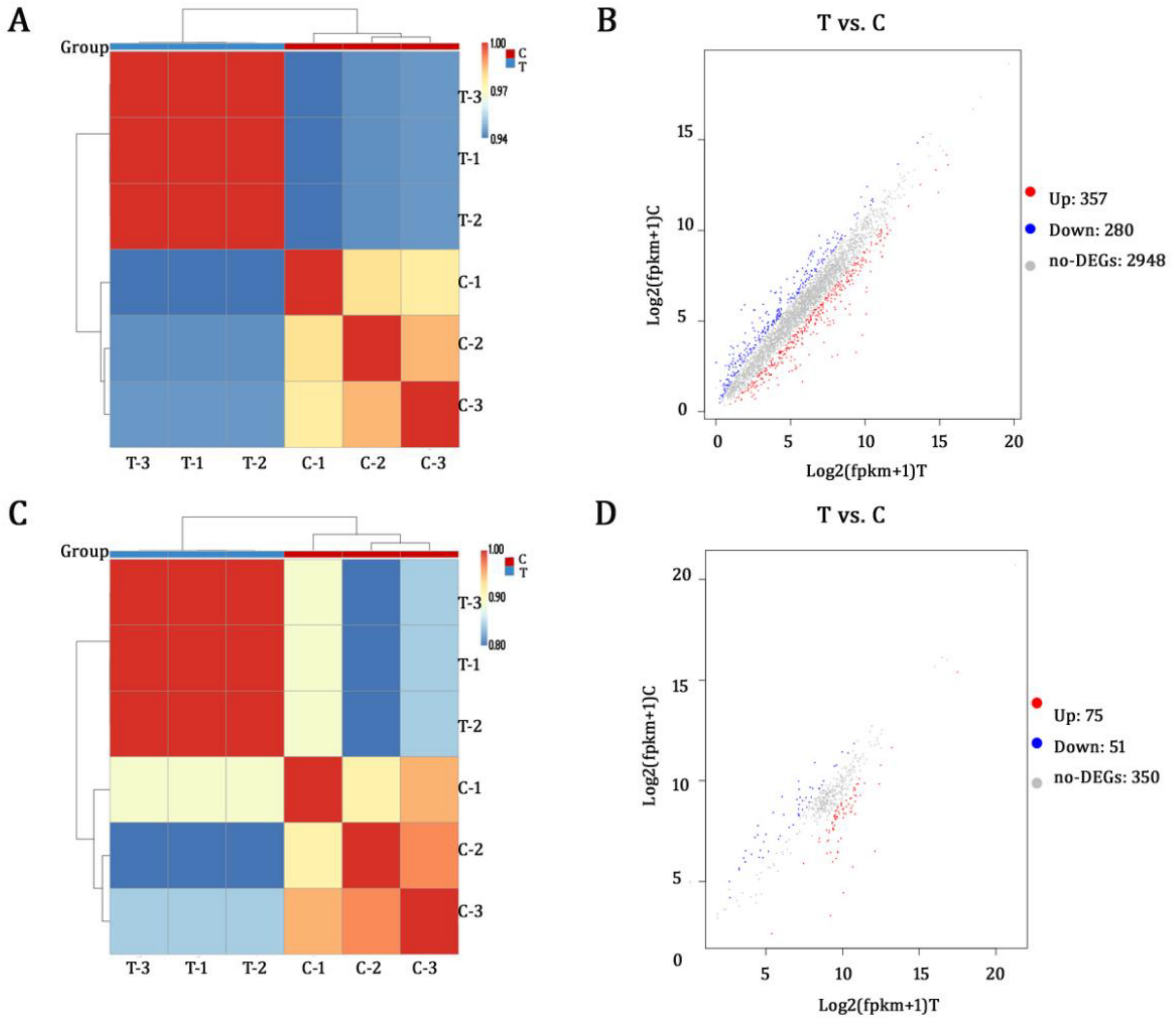
Overview of the acetone extract of *M. paniculata* leaf on *E. coli* gene expression: **(A)** heatmap of six samples from two groups based on mRNA correlation analysis; **(B)** a scatterplot of differentially expressed mRNAs; **(C)** heatmap of six samples from two groups based on sRNA correlation analysis; **(D)** a scatterplot of differentially expressed sRNAs. C, control; T, treatment; AEML, acetone extract of *M. paniculata* leaf; T1**–**3, samples treated with 200 μg/mL AEML; C1**–**3, samples without addition of AEML.

Principal component analysis (PCA) assessed the clustering of samples and showed clear separation between the control (C) and treated (T) groups. The Pearson’s correlation analysis among the three biological replicates of each experimental group revealed high correlation coefficients (R^2^ > 0.94 for mRNA, R^2^ > 0.81 for sRNA) (**Figures 2A, C**). Under the thresholds of FDR<0.05 and |log2FC|≥1, there were 357 upregulated and 280 downregulated mRNAs (**Figure 2B**) and 75 upregulated and 51 downregulated sRNAs (**Figure 2D**). The distinct clustering of the two groups and the large number of differentially expressed genes (DEGs) indicate obvious impacts of AEML on *E. coli* gene transcription.

The GO enrichment analysis suggests significant impacts of AEML on *E. coli* function (**Supplementary Figure S1**). In biological processes, much more genes involved in cell killing, nitrogen utilization, and signaling were upregulated. As shown in **Figure 1C**, cell-killing processes often involve the crossing or disrupting cellular membranes. By modifying membrane composition or inducing oxidative damage, antimicrobial agents can efficiently kill bacteria (Wang et al., 2017).

In cellular components, more genes associated with the extracellular region, host cellular components, and the nucleoid were upregulated. In molecular functions, enriched upregulated genes were involved in antioxidant activity and toxin activity, whereas downregulated genes were associated with molecular transducer activity and translation regulator activity.

**Figure 3:**
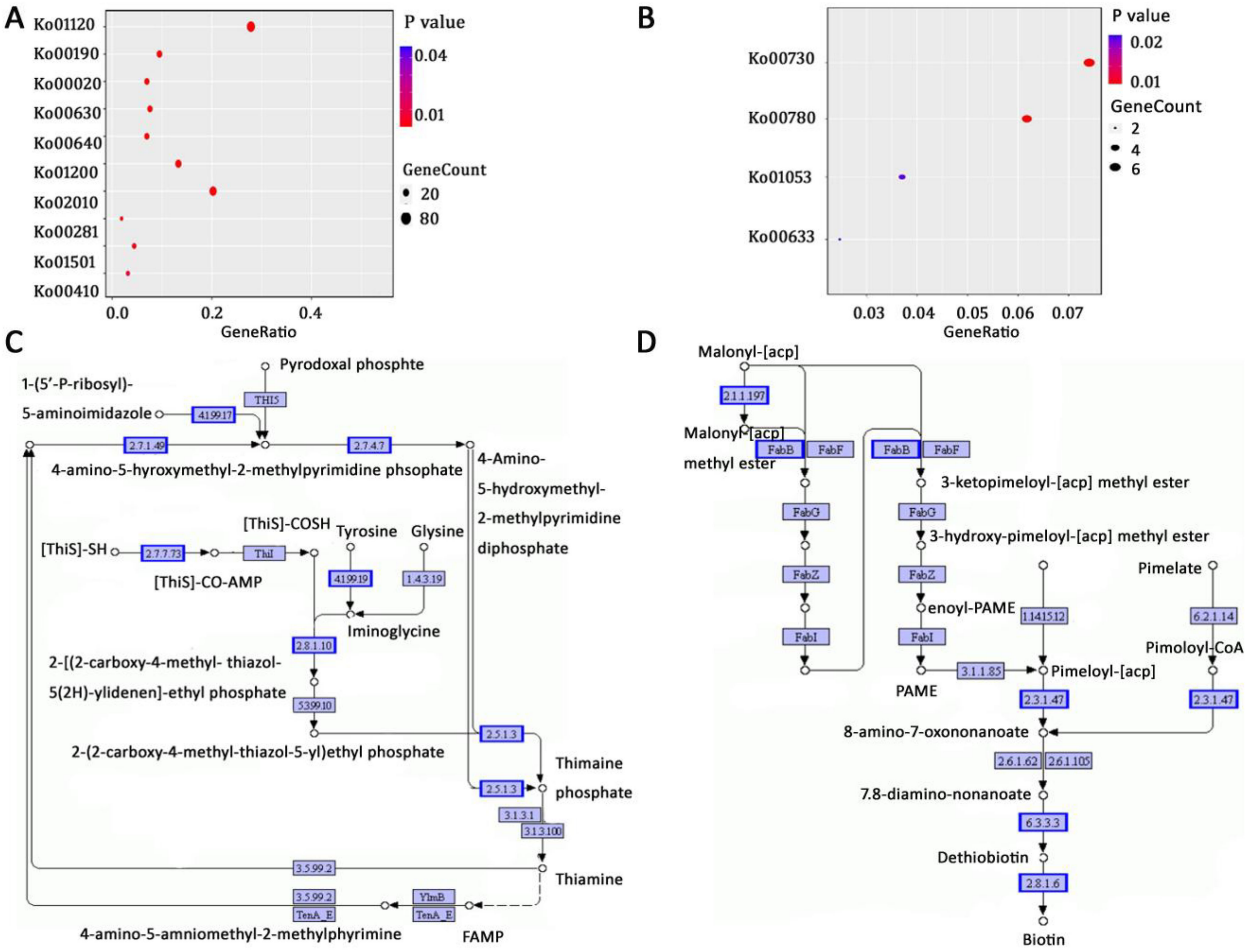
The KEGG enrichment and pathway analysis of *E. coli* differentially expressed genes: **(A)** a dotplot of the top ten upregulated pathways; **(B)** dotplot of the significantly downregulated pathways; **(C)**, thiamine metabolism pathway; **(D)** biotin metabolism pathway. Boxed genes with blue outlines are significantly downregulated.

The KEGG enrichment analysis showed that many upregulated DEGs were enriched in diverse pathways, including microbial metabolism in diverse environments (Ko01120), oxidative phosphorylation (Ko00190), and the citrate cycle (Ko00020) (**Figure 3A**). Oxidative phosphorylation is responsible for the energy coupling and ATP synthesis via electron carriers and protein complexes, while the citrate cycle supports major energy production and biosynthesis in microorganisms (Nath and Villadsen, 2015; Martinez et al., 2024).

In contrast, downregulated DEGs were significantly enriched in only four pathways: thiamine metabolism (Ko00730), biotin metabolism (Ko00780), biosynthesis of siderophore group nonribosomal peptides (Ko01053), and nitrotoluene degradation (Ko00633) (**Figure 3B**). Thiamine and biotin are both B vitamins essential for health and energy metabolism. As indispensable cofactors for nearly all organisms, thiamine and biotin are required for carbohydrate and branched-chain amino acid metabolic enzymes (Du et al., 2011), including carboxylases, decarboxylases, and transcarboxylases—enzymes involved in intermediary metabolism such as gluconeogenesis, fatty acid synthesis, and amino acid metabolism (Sirithanakorn and Cronan, 2021).

In *E. coli*, the *thiCEFGSH* operon is responsible for thiamine *de novo* synthesis. In the presence of AEML, the downregulated genes in thiamine metabolism included *thiC, thiD, thiE, thiF, thiG*, and *thiH* (**Figure 3C**). It was previously reported that the opportunistic pathogen *Pseudomonas aeruginosa* exploits bacterial biotin synthesis pathways to enhance infection (Shi et al., 2023). Therefore, interfering with *de novo* biotin synthesis has been considered as an attractive antimicrobial strategy for certain recalcitrant infections. We observed that several genes in biotin metabolism—including *bioB, bioC, bioD, bioF*, and *tabB*—were downregulated (**Figure 3D**). Collectively, these results suggest that the genes mentioned above may represent novel molecular targets for the antimicrobial effects of AEML.

The top ten downregulated and top ten upregulated genes were further summarized by sorting their log_2_FC values. As shown, the expression of gene *DR76_RS29455* appears to be completely inhibited by AEML, as no reads were detected in the T group (**Supplementary Table S4**). Among the upregulated genes, *nemR* and *btsT* ranked the highest (**Supplementary Table S5**). NemR belongs to the TetR family of HTH-type DNA-binding transcription factors. Together with *NemA*, it plays an important role in *E. coli* survival in the presence of the toxic compounds (Umezawa et al., 2008). Methylglyoxal (MG), a highly reactive by-product of glycolysis, is known to induce the *nemRA* operon when cells are exposed to growth-inhibitory concentrations (Ozyamak et al., 2013). In fact, MG is also the major component underlying the antibacterial activity of manuka honey (Majtan, 2011). These results suggest that AEML might lead to increased MG synthesis in *E. coli*, thereby contributing to growth inhibition. For btsT, a pyruvate/H^+^ symporter, it facilitates the uptake of pyruvate from the medium to support *E. coli* growth and survival of *E. coli* when nutrients are limited (Kristoficova et al., 2017). Pyruvate also promotes resuscitation when *E. coli* enters a viable but nonculturable state under adverse environmental conditions (Vilhena et al., 2019). Therefore, it is possible that *E. coli* may employ a similar strategy when exposed to AEML.

In addition, as a type of regulatory RNA, small RNAs (sRNAs) of 50–500 nucleotides that do not encode proteins are typically located in noncoding regions between two protein-coding genes or derived from the 5′ or 3′ untranslated regions of mRNA (Papenfort and Storz, 2024). These sRNAs can regulate gene expression through various mechanisms, including mRNA degradation, translation repression, transcription repression, and transcription stabilization. They play an important roles in bacterial growth, metabolism, stress responses, and pathogenicity (Gottesman, 2025).

Using RIsearch, only yields 65 target genes were predicted, whereas RNAhybrid yielded many more target predictions for the 476 sRNAs—identifying putative microRNA-like target sites of microRNAs (miRNAs) in 3′UTRs (**Supplementary Figure S2**). Among the ten most downregulated sRNAs, predicted_RNA298 and predicted_RNA57 ranked highest (**Supplementary Table S6**). For the ten most upregulated sRNAs, predicted_RNA335 and predicted_RNA336 ranked highest (**Supplementary Table S7**). Predicted structures for these four sRNAs are shown in **Supplementary Figure S3**.

Among these twenty sRNAs, only one downregulated sRNA (predicted_RNA128) had a single predicted target gene—*DR76_RS05075*—confirmed by both software tools. This gene encodes GadE, an acid resistance transcriptional activator. GadE protein is a key regulator that modulates *fliC* gene transcription and flagellar motility in *E. coli* (Schwan et al., 2020). However, the other sRNAs either had multiple targets or no target was predicted targets or no targets at all, making regulatory interpretation difficult.

### 3.3 Chemical profile of AEML detected by LC-MS/MS

According to a recent review on *M. paniculata*, 720 compounds have been identified from *M. paniculata*, including flavonoids, coumarins, alkaloids, sterols, phenylpropenols, organic acids, spirocyclopentenones, and volatile oils (Qi et al., 2024). In this study, we detected 1,363 metabolites under the positive ion mode and 282 metabolites under the negative ion mode using the UPLC-MS/MS detection platform and a self-built database analysis under positive and negative ion models with six replicates for each mode. This number is much higher than that reported in a recent study, which identified only 212 compounds were identified from *M. paniculata* dry leaves and twigs using a quantitative chemomics strategy based on LC-MS (Liang et al., 2024).

**Figure 4:**
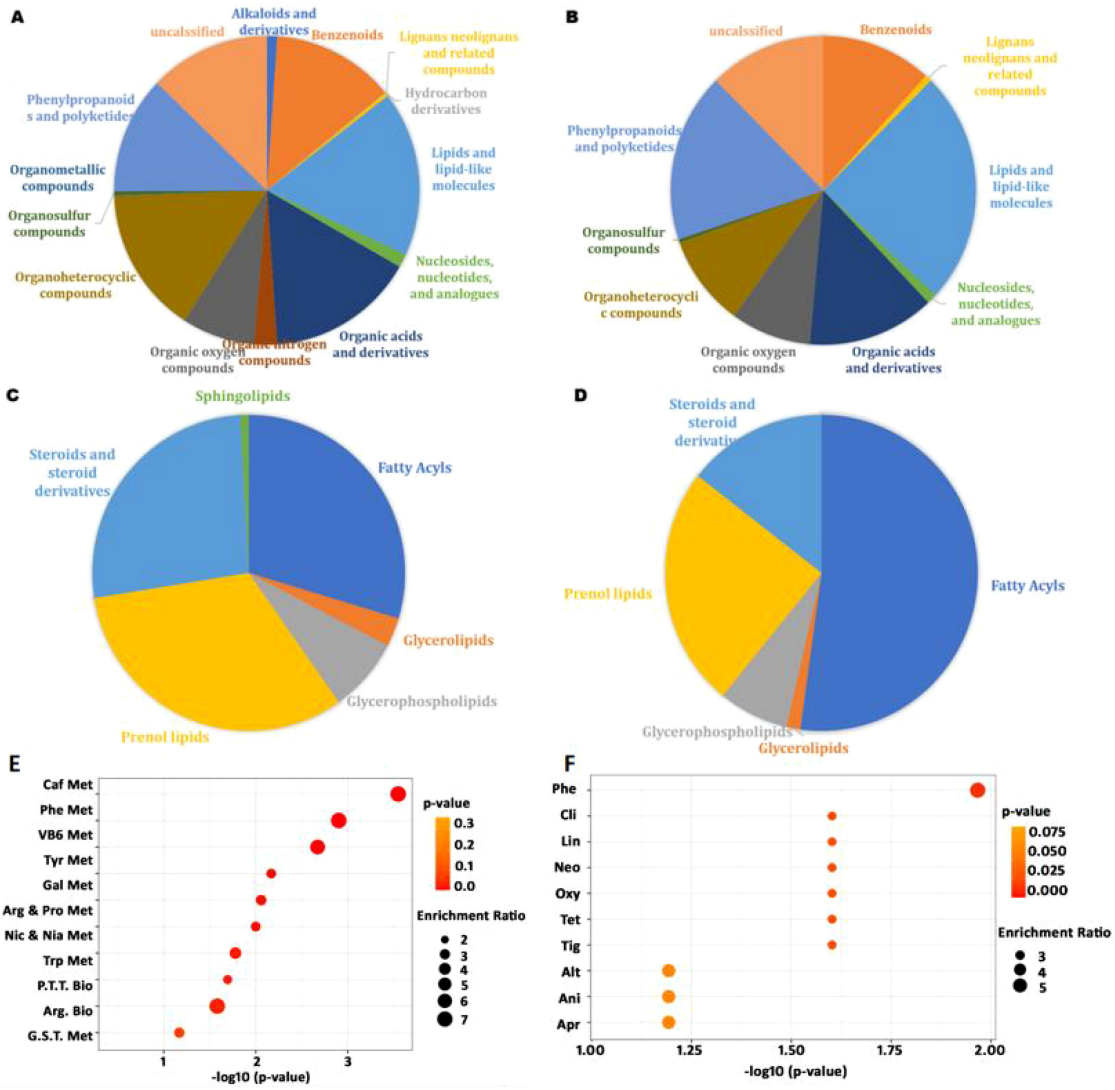
The composition of *M. paniculata* leaf metabolites detected by LC-MS/MS and their enrichment: **(A)** the superclass of 1,363 detected metabolites under the positive ion model; **(B)** the superclass of 282 detected metabolites under the negative ion model; **(C)** the class of 236 lipids and lipid-like molecules under the positive ion model; **(D)** the class of 69 lipids and lipid-like molecules under the negative ion model; **(E)** metabolites enriched in KEGG pathways; **(F)** metabolites enriched in drug pathways from SMPDB. The top ten enriched pathways were included in the bubble charts. In panel **E**: Caf Met, caffeine metabolism; Phe Met, phenylalanine metabolism; VB6 Met, vitamin B6 metabolism; Tyr Met, tyrosine metabolism; Gal Met, galactose metabolism; Arg & Pro Met, arginine and proline metabolism; Nic & Nia Met, nicotinate and nicotinamide metabolism; Try Met, tryptophan metabolism; P.T.T. Met, phenylalanine, tyrosine, and tryptophan metabolism; Arg Bio, arginine biosynthesis. In panel **F**: Phe, phenindione; Cli, clindamycin; Lin, lincomycin; Neo, neomycin; Oxy, oxytetracycline; Tet, tetracycline; Tig, tigecycline; Alt, alteplase; Ani, anistreplase; Apr, aprotinin.

As shown in **Figures 4A, B**, the detected metabolites under both positive and negative ion modes included benzenoids, lignans and neolignans, lipids and lipid-like molecules, nucleosides and nucleotides, and analogues, organic acids and derivatives, organic oxygen compounds, organoheterocyclic compounds, organosulfur compounds, phenylpropanoids, and polyketides, as defined by SuperClass. Among them, lipids and lipid-like molecules constituted the largest category (**Supplementary Table S8**). Within this superclass of lipids and lipid-like molecules, fatty acyls, prenol lipids, steroids, and steroid derivatives were the most abundant classes (**Figures 4C, D**). The top ten most abundant metabolites under both ionization modes included adenosine, arginine, trigonelline, and others (**Supplementary Table S9**). Additionally, phenols, carboxylic acids and derivatives, nucleotides and derivatives, coumarins and derivatives, and flavonoids were also abundant, consistent with previous findings (Yohanes et al., 2023).

In the literature, various antimicrobial agents have been employed to combat pathogenic microbes. Within the superclass of phenylpropanoids and polyketides, coumarins and cinnamic acids (and their derivatives) are the major metabolites. Coumarins exhibit antimicrobial activity against a variety of pathogens, largely due to their ability to interact with diverse enzymes and receptors in living organisms (Annunziata et al., 2020). In our study, as many as 28 coumarins and derivatives were detected under the positive ion mode, classified into coumarin glycosides, furanocoumarins, hydroxycoumarins, and pyranocoumarins subclasses (**Supplementary Table S10**).

Cinnamic acids and derivatives included one cinnamic acid, one cinnamic acid ester, and 17 hydroxycinnamic acids and derivatives. Some hydroxycinnamic acid derivatives exhibit antibacterial and antimalarial activity (Ruwizhi and Aderibigbe, 2020). Sesquiterpene hydrocarbons—particularly caryophyllene—have previously been identified as the major compounds of the volatile oils and were responsible for the antimicrobial activity in *M. paniculata* leaves (Saikia et al., 2021). Here, we detected 20 sesquiterpenoids, including caryophyllene oxide (**Supplementary Table S11**).

Among the metabolites identified under the positive and negative ion models, there are 823 and 153 compounds, respectively, could be retrieved in the KEGG database. According to the KEGG compound search, many metabolites had biological roles, including amino acids and fat- or water-soluble vitamins. Notably, *M. paniculata* also synthesizes other amino acids, such as D-arginine and γ-aminobutyric acid. D-amino acids can ameliorate various oxidative stress and modulate the gut microbiota, thereby protecting several organs such as the bowel and stomach (Ikeda et al., 2022).

Metabolites were significantly enriched in caffeine metabolism, phenylalanine metabolism, vitamin B6 metabolism, tyrosine metabolism, and phenylalanine–tyrosine–tryptophan biosynthesis, among others (**Figure 4E**). Phenylalanine is an essential amino acid required for the biosynthesis of other amino acids and participates in the structure and function of many proteins and enzymes (Egbujor et al., 2024). Vitamin B6 is a coenzyme involved in more than 150 biochemical reactions, including the metabolism of carbohydrates, lipids, amino acids, and nucleic acids, and participates in cellular signaling (Stach et al., 2021).

Based on drug pathways from the Small Molecule Pathway Database (SMPDB), we find that the metabolites were mainly enriched in pathways related to phenindione, clindamycin, lincomycin, neomycin, oxytetracycline, tetracycline, and tigecycline action pathways (**Figure 4F**). Phenindione is a well-known anticoagulant that inhibits vitamin K reductase and prevents harmful blood clots (Nikolova et al., 2023). Clindamycin and lincomycin are lincosamide antibiotics used to treat Gram-positive cocci and bacilli, as well as Gram-negative cocci and some other organisms, including staphylococcal, streptococcal, and anaerobic bacterial infections (Spížek and Řezanka, 2017). Neomycin, oxytetracycline, tetracycline, and tigecycline are also antibiotics widely used clinical antibiotics. These data suggest that the broad-range antimicrobial effect of *M. paniculata* may also be partially attributed to the synthesis of various antibiotic-like metabolites.

Antibacterial properties against human pathogens have also been frequently associated with phenols and flavonoids, likely through effects on cell membrane fluidity (Gautam et al., 2012). A total of 23 kinds of phenols were detected, including mainly benzenediols and methoxyphenols (**Supplementary Table S12**). In contrast to a previous report by Menezes et al., which identified ellagic acid as the principal phenolic component by HPLC-DAD, our study showed that trans-3,5-dimethoxy-4-hydroxycinnamaldehyde (also known as sinapaldehyde) is the most abundant phenol in the leaf extract of *M. paniculata* leaf extract (Menezes et al., 2015). The second most abundant phenol was coniferyl alcohol, which is a flavor compound and lignin precursor. Lignin particles possess antioxidant and antimicrobial properties (Ali et al., 2024). One known antimicrobial mechanism of phenolic compounds is disruption of cell peptidoglycan or damage of the cell membrane integrity, causing the leakage of intracellular constituents such as proteins, glutamate, potassium, and phosphate from bacteria (Chen et al., 2024).

Flavonoids and isoflavonoids exhibit antimicrobial activity by inhibiting nucleic acid synthesis, cytoplasmic membrane function, and energy metabolism (Shamsudin et al., 2022). Not surprisingly, many flavonoids and isoflavonoids were present in the leaf of *M. paniculata*: specifically, 124 flavonoids and 16 isoflavonoids were detected. Besides the cell membrane effects, DNA gyrase has also been identified as an important antibacterial target of plant flavonoids against Gram-negative bacteria (Yan et al., 2024).

### 3.4 Antibiotics in the acetone extract of *M. paniculata* leaf

Serious infections and increasing resistance to current antibiotics highlight the urgent need to discover natural antimicrobial compounds. To some extent, it is out of our expectation that many widely recognized antibiotics were detected in the AEML. As summarized in **Table 1**, only three of the ten common classes of antibiotics—macrolides, glycopeptides, and carbapenems—were absent (https://www.drugs.com/article/antibiotics.html). The remaining seven classes—penicillins, tetracyclines, cephalosporins, fluoroquinolones, lincosamides, sulfonamides, and aminoglycosides—were represented by 19 different antibiotics are presented in the extract of *M. paniculata* leaf. These antibiotics act through inhibition of cell wall synthesis, DNA and protein synthesis, and folic acid synthesis.

**Table 1:** Detail information of some antibiotics from the acetone extract of *M. paniculata* leaf detected by LC-MS/MS.

No.	ID	Name	KEGG No.	Antibiotic class	Function
1	M379T415_1	Carbenicillin	None	Penicillin	Inhibit cell wall synthesis
2	M401T473	α-carboxybenzylpenicillin	C06869		
3	M333T242	Ampicillin	C06574		
4	M445T327	Tetracycline	C06570	Tetracycline	Inhibit protein synthesis (anti-30S ribosomal subunit)
5	M426T377	Oxytetracycline	C06624		
6	M569T36	Tigecycline	C12012		
7	M364T301	Cefadroxil	C06878	Cephalosporin	Inhibit cell wall synthesis
8	M463T489	Cefamandole	C06879		
9	M547T401	Ceftazidime	C06889		
10	M320T35_1	Norfloxacin	C06687	Fluoroquinolone	Inhibit DNA synthesis
11	M366T9	N-desmethyldanofloxacin	None		
12	M386T446	Sarafloxacin	None		
13	M332T309_1	Ciprofloxacin	C05349		
14	M215T33_1	Nalidixic acid	C05079		
15	M429T246	Lincomycin	C06812	Lincomycin	Inhibit protein synthesis (anti-50S ribosomal subunit)
16	M425T218	Clindamycin	C06914		
17	M381T419	Sulfasalazine	C07316	Sulfonamide	Inhibit folic acid synthesis
18	M405T321	Sulfinpyrazone	C07317		
19	M615T249	Neomycin A	C01737	Aminoglycopeptide	Inhibit protein synthesis (anti-30S ribosomal subunit)

Further validation using specific antibodies confirmed the presence of ampicillin, norfloxacin, doxorubicin, and tigecycline in *M. paniculata* (**Supplementary Figure S5**).

In addition, 12 other antibiotics were detected in the AEML by LC-MS (**Supplementary Table S11**). For example, fusidic acid inhibits protein synthesis by binding elongation factor G, thereby blocking peptide translocation and ribosome disassembly, while oligomycins inhibit ATP synthase and induce significant G1-phase cell cycle arrest (Fernandes, 2016; Feng et al., 2024). Of particular significance, chrysomycin A exhibits potent anti-tuberculosis activity (MIC = 0.4 μg/mL) against multidrug-resistant strains (Wu et al., 2020). Tuberculosis remains a life-threatening disease, causing an estimated 10 million new infections and 1.8 million deaths annually, primarily in underdeveloped countries. Considering that the drug Sanjiu Weitai Granule, which uses *M. paniculata* as a major component, is commonly used for treating gastric diseases, chrysomycin A may play an important role in ameliorating infectious gastritis.

## 4 Conclusion

The extracts of *M. paniculata* leaf moderately inhibited both Gram-positive bacteria *(S. aureus* and *St. porcinus*) and Gram-negative bacteria (*Sa. typhimurium* and *E. coli*), although the inhibition effect varied depending on the solvent and species. The AEML inhibited the growth of *E. coli* with a MIC of 400 μg/mL, partially due to disruption of the cell wall and membrane. The AEML regulated the gene expression, and a large number of DEGs were enriched in oxidative phosphorylation and the citrate cycle in *E. coli*. The most downregulated pathways were thiamine metabolism and biotin metabolism, suggesting that AEML may decrease the synthesis of essential cofactors in *E. coli*.

More than 1,000 metabolites in AEML were identified by LC-MS/MS. Among them, many were potential antimicrobial agents, including phenols, flavonoids, isoflavonoids, coumarins, cinnamic acids, sesquiterpenoids, and their derivatives. Many metabolites of *M. paniculata* were enriched in caffeine metabolism, phenylalanine metabolism, and vitamin B6 metabolism in the KEGG pathways. In the drug pathways, these metabolites were mainly enriched in phenindione, clindamycin, lincomycin, neomycin, oxytetracycline, tetracycline, and tigecycline action pathways. For the first time, more than 30 antibiotics—both common and potentially novel natural products—were detected by LC-MS. In conclusion, this study provides novel molecular insights into the antimicrobial effects of *M. paniculata* leaf extracts.

## Data Availability

The datasets presented in this study can be found in online repositories. The names of the repository/repositories and accession number(s) can be found in the article/**Supplementary Material**.
