# Supplementary material for: Transcriptomic and metabolomic insights into the antimicrobial mechanisms of Murraya paniculata (L.) Jack leaf extract
Source: Front Plant Sci. 2025 Dec 2;16:1717793. doi: 10.3389/fpls.2025.1717793 (PMC12705546; doi:10.3389/fpls.2025.1717793)
Supplement: Supplementary file 1 [file DataSheet1.docx]

Supplementary Information

Transcriptomic and metabolomic insights into the antimicrobial mechanisms of *Murraya paniculata* (L.) Jack leaf extract

Qing Ma^1, 3^, Lin Zhang^2, 3^, Azhen Nie^4^, Yini Shi^4^, Zhongqiu Liu^1^, Rongrong Zhang^1, *^, Zhongke Sun ^4, *^

1. International Institute for Translational Chinese Medicine, School of Pharmaceutical Sciences, Guangzhou University of Chinese Medicine, Guangzahou, 51006, China

2. Guangzhou Medical University, State Key Laboratory of Respiratory Disease，The First Affiliated Hospital of Guangzhou Medical University，Guangzhou 510120, China

3. China Resources Sanjiu Medical & Pharmaceutical Co., Ltd, Shenzhen, 518110, China

4. School of Biological Engineering, Henan University of Technology, Zhengzhou 450001, China

*, corresponding authors

Prof. Rongrong Zhang, Email: zrr586@gzucm.edu.cn

Post address: No.232, Waihuan Road, Guangzhou University of Chinese Medicine, Guangzhou 51006, Guangdong Province, China

Prof. Zhongke Sun, Email: [sunzh@daad-alumni.de](mailto:sunzh@daad-alumni.de);

ORCID: <https://orcid.org/0000-0002-9784-9769>;

Post address: No.100, Lianhua Road, Zhengzhou 450001, Henan Province, China

Table S1 The antimicrobial effect of different extracts of *M. paniculata* leaf measured by the agar diffusion

| **Strains/Solvents** | ***S. aureus*** | ***St. porcinus*** | ***Sa. typhimurium*** | ***E. coli*** |
| --- | --- | --- | --- | --- |
| Acetone | 5mm | 3mm | 3mm | 3mm |
| Methanol | 6mm | - | 2mm | - |
| Ethanol | 2mm | 4mm | 5mm |  |
| Hexane | - | - | - | - |
| Water | - | - | 2mm | - |

Note: *S. aureus, Staphylococcus aureus* ATCC 6538*; St. porcinus, Streptococcus porcinus* GDMCC NO.1.1044*; Sa. typhimurium, Salmonella enterica* subsp. typhimurium ATCC 14028*; E. coli, Escherichia coli* ETEC GDMCC NO.1.4025.

Table S2 Summary of RNA-seq clean data after quality control

| **Sample_ID** | **Total_Reads** | **Total_Bases** | **Q20_Bases** | **Q30_Bases** | **Q20_Rate(%)** | **Q30_Rate(%)** | **GC_Content(%)** |
| --- | --- | --- | --- | --- | --- | --- | --- |
| C-1 | 49,727,956 | 6,449,314,175 | 6,416,817,896 | 6,313,835,159 | 99.4961 | 97.8993 | 49.84 |
| C-2 | 45,691,860 | 6,078,159,858 | 6,046,221,682 | 5,945,077,448 | 99.4745 | 97.8105 | 49.7071 |
| C-3 | 52,993,012 | 6,807,934,117 | 6,775,430,397 | 6,670,483,147 | 99.5226 | 97.981 | 49.6408 |
| T-1 | 51,648,934 | 6,589,750,030 | 6,556,802,586 | 6,451,550,648 | 99.5 | 97.9028 | 51.7159 |
| T-2 | 51,648,934 | 6,589,750,030 | 6,556,802,586 | 6,451,550,648 | 99.5 | 97.9028 | 51.7159 |

Note: Sample_ID: Sample name; Total_Reads: Count the original sequence data, with four rows as a unit, to tally the number of sequencing reads per file; Total_Bases: Total sequencing base count, generally equal to sequence data * sequence length; Q20_Bases: Total number of bases with Phred quality >=20; Q30_Bases: Total number of bases with Phred quality >=30; Q20_Rate: Percentage of bases with Phred quality >=20 relative to the total base count; Q30_Rate: Percentage of bases with Phred quality >=30 relative to the total base count; GC content: Calculate the percentage of total bases that are G and C

Table S3 The number of annotated genes in different databases after RNA sequencing

| **database** | **No.** |
| --- | --- |
| GO | 3849 |
| KEGG | 3211 |
| PFAM | 4019 |
| SWISSPROT | 4285 |
| STRING | 3338 |
| NR | 5010 |
| Annotated in all databases | 2145 |

Note: NR, non-redundant; STRING, Search Tool for the Retrieval of Interacting Genes; GO, Gene Ontology; COG Clusters of Orthologous Groups of proteins; KEGG, Kyoto Encyclopedia of Genes and Genomes

Table S4 The top ten downregulated mRNAs in *E. coli* after treatment with acetone extract of *M. paniculata* leaf

| **gene_id** | **expression_T** | **expression_C** | **log2FC(T/C)** | **gene_name** |
| --- | --- | --- | --- | --- |
| DR76_RS29455 | 0 | 5.53902 | -9.1161 | *DR76_RS29455* |
| DR76_RS29150 | 2.2328 | 48.0583 | -4.4217 | *ynfR* |
| DR76_RS17120 | 2.7644 | 57.9318 | -4.3843 | *ynfN* |
| DR76_RS13380 | 1.0204 | 11.0988 | -3.4305 | *araB* |
| DR76_RS10545 | 4.7671 | 47.2769 | -3.3072 | *thiC* |
| DR76_RS22580 | 0.2234 | 2.2502 | -3.2757 | *speF* |
| DR76_RS12840 | 5.0429 | 47.758 | -3.2409 | *fhuF* |
| DR76_RS12835 | 0.8694 | 7.8293 | -3.1561 | *bglJ* |
| DR76_RS10530 | 1.2219 | 10.3334 | -3.0697 | *thiS* |
| DR76_RS10535 | 11.7607 | 98.7141 | -3.0682 | *thiF* |

Table S5 The top ten upregulated mRNAs in *E. coli* after treatment with acetone extract of *M. paniculata* leaf

| **gene_id** | **expression_T** | **expression_C** | **log2FC(T/C)** | **gene_name** |
| --- | --- | --- | --- | --- |
| DR76_RS16625 | 671.0876 | 8.6504 | 6.2760 | *nemR* |
| DR76_RS12770 | 899.1073 | 17.7030 | 5.6656 | *btsT* |
| DR76_RS17370 | 283.8583 | 7.7571 | 5.1917 | *marA* |
| DR76_RS17375 | 220.4276 | 7.1996 | 4.9343 | *marR* |
| DR76_RS16620 | 1068.2010 | 41.1786 | 4.6968 | *nemA* |
| DR76_RS17365 | 54.2809 | 2.1665 | 4.6406 | *marB* |
| DR76_RS21805 | 500.0886 | 39.9529 | 3.6455 | *ybjC* |
| DR76_RS22080 | 150.9476 | 13.0301 | 3.5331 | *cecR* |
| DR76_RS00850 | 1072.1560 | 96.0258 | 3.4808 | *inaA* |
| DR76_RS09140 | 49.2460 | 4.9390 | 3.3151 | *ygeW* |

Table S6 The top ten downregulated sRNAs and their potential target genes in *E. coli* after treatment with acetone extract of *M. paniculata* leaf

| **gene_id** | **expression_T** | **expression_C** | **log2FC(T/C)** | **RNAhybrid target genes (No.)** | **RIsearch target gene** |
| --- | --- | --- | --- | --- | --- |
| predicted_RNA298 | 21.2449 | 216.4304 | -3.3481 | 115 |  |
| predicted_RNA57 | 64.9250 | 590.5338 | -3.1850 | - | - |
| predicted_RNA82 | 43.2833 | 339.7679 | -2.9724 | DR76_RS00955; DR76_RS00965 | - |
| predicted_RNA393 | 18.4495 | 144.1378 | -2.9651 | 115 | - |
| predicted_RNA179 | 11.7406 | 90.0568 | -2.9383 | 115 | - |
| predicted_RNA96 | 20.9654 | 157.1811 | -2.9058 | 42 | - |
| **predicted_RNA128** | **139.6603** | **1002.7020** | **-2.8438** | **5** | **DR76_RS05075** |
| predicted_RNA59 | 10.7057 | 70.3614 | -2.7153 | *DR76_RS00015* | - |
| predicted_RNA77 | 290.0686 | 1899.9642 | -2.7115 | 395 | - |
| predicted_RNA98 | 5.1213 | 30.7638 | -2.5843 | 30 | - |

Table S7 The top ten upregulated sRNAs and their potential target genes in *E. coli* after treatment with acetone extract of *M. paniculata* leaf

| **gene_id** | **expression_T** | **expression_C** | **log2FC(T/C)** | **RNAhybrid target genes (No.)** | **RIsearch target gene** |
| --- | --- | --- | --- | --- | --- |
| predicted_RNA335 | 594.6537 | 8.7982 | 6.0771 | - | - |
| predicted_RNA336 | 1062.2447 | 20.4695 | 5.6968 | 52 | - |
| predicted_RNA337 | 4434.7893 | 88.7934 | 5.6421 | 159 | - |
| predicted_RNA449 | 1619.2979 | 51.2727 | 4.9808 | 116 | - |
| predicted_RNA8 | 763.2605 | 69.1320 | 3.4646 | - | - |
| predicted_RNA407 | 655.3295 | 61.8863 | 3.4043 | 19 | - |
| predicted_RNA552 | 820.1493 | 84.2920 | 3.2823 | 55 | - |
| predicted_RNA479 | 40.4004 | 4.2797 | 3.2358 | 31 | - |
| predicted_RNA559 | 822.3304 | 88.1670 | 3.2213 | 62 | - |
| predicted_RNA501 | 598.5057 | 85.0846 | 2.8142 | 507 | - |

Table S8 The chemical profile of the acetone extract of *M. paniculata* leaf

| **SuperClass** | **POS** | **NEG** |
| --- | --- | --- |
| Alkaloids and derivatives | 15 | 0 |
| Benzenoids | 177 | 33 |
| Hydrocarbon derivatives | 1 | 0 |
| Lignans neolignans and related compounds | 5 | 2 |
| Lipids and lipid-like molecules | 236 | 69 |
| Nucleosides, nucleotides, and analogues | 19 | 3 |
| Organic acids and derivatives | 214 | 38 |
| Organic nitrogen compounds | 33 | 0 |
| Organic oxygen compounds | 105 | 24 |
| Organoheterocyclic compounds | 210 | 27 |
| Organometallic compounds | 1 | 0 |
| Organosulfur compounds | 5 | 1 |
| Phenylpropanoids and polyketides | 168 | 50 |
| uncalssified | 174 | 35 |
|  |  |  |
| **Class of lipids and lipid-like molecules** | **POS** | **NEG** |
| Fatty Acyls | 70 | 36 |
| Glycerolipids | 7 | 1 |
| Glycerophospholipids | 18 | 5 |
| Prenol lipids | 76 | 17 |
| Steroids and steroid derivatives | 63 | 10 |
| Sphingolipids | 2 | 0 |

Table S9 The top ten metabolites identified under both positive and negative ion models in the acetone extract of *M. paniculata* leaf

| **ID** | **m/z** | **rt(s)** | **Name** | **Model** | **%** |
| --- | --- | --- | --- | --- | --- |
| M268T192_1 | 268.10392 | 191.737 | Adenosine | + | 6.6965 |
| M116T336 | 116.07049 | 335.742 | DL-arginine | + | 5.4843 |
| M138T314_2 | 138.05496 | 313.579 | Trigonelline | + | 4.9921 |
| M593T64_3 | 593.27559 | 63.6854 | Pheophorbide a | + | 4.8921 |
| M189T31_1 | 189.05447 | 31.3561 | Osthole | + | 4.7788 |
| M104T296_3 | 104.10687 | 296.415 | Choline | + | 4.2818 |
| M265T283_2 | 265.15456 | 282.824 | Feruloyl putrescine | + | 3.9302 |
| M243T31 | 243.10128 | 31.4824 | beta-lapachone | + | 3.8592 |
| M175T549 | 175.11878 | 549.2075 | Arginine | + | 3.0497 |
| M259T33_1 | 259.09631 | 32.5734 | Murrayone | + | 2.7562 |
| M243T31 | 243.10128 | 31.4824 | Biotin | - | 33.5057 |
| M259T33_1 | 259.09631 | 32.5734 | Lumichrome | - | 24.2203 |
| M219T31_1 | 219.06508 | 31.3561 | 5-Hydroxytryptophan | - | 5.8635 |
| M439T453_4 | 439.14195 | 453.427 | Aminopterin | - | 3.4037 |
| M207T134 | 207.06513 | 134.365 | Trans-3,5-dimethoxy-4-hydroxycinnamaldehyde | - | 3.2067 |
| M389T101 | 389.08668 | 100.867 | Scaposin | - | 2.4722 |
| M389T158 | 389.08677 | 157.56 | 4',5,7-trihydroxy 3,3',6,8-tetramethoxyflavone | - | 2.2892 |
| M399T39 | 399.16471 | 39.1898 | [6-hydroxy-1-[2-(4-morpholinyl)ethyl]-1h-indol-3-yl]-1-naphthalenyl- | - | 1.5364 |
| M203T330 | 203.10281 | 329.978 | Ser-Val | - | 1.4434 |
| M351T380_2 | 351.12608 | 379.571 | beta.-Estradiol 3-sulfate | - | 0.9931 |

Table S10 Coumarins and cinnamic acids and their derivatives in the acetone extract of *M. paniculata* leaf

| **Class** | **Subclass** | **Metabolites No.** | **Representative antimicrobial agent** |
| --- | --- | --- | --- |
| Coumarins and derivatives | Coumarin glycosides | 5 | Rutarin |
|  | Furanocoumarins | 9 | Bergaptol |
|  | Hydroxycoumarins | 8 | Scopoletin |
|  | Pyranocoumarins | 6 | Luvangetin |
| Cinnamic acids and derivatives | Cinnamic acid | 1 | Trans-cinnamic acid |
|  | Cinnamic acid ester | 1 | Picroside i |
|  | Hydroxycinnamic acids and derivatives | 17 | P-coumaric acid |

Table S11 Sesquiterpenoids in the acetone extract of *M. paniculata* leaf

| **ID** | **m/z** | **rt(s)** | **Name** |
| --- | --- | --- | --- |
| M499T187 | 499.18091 | 186.6005 | Gossypol |
| M245T52 | 245.11705 | 52.0682 | 2-cis-4-trans-abscisic acid |
| M233T34 | 233.15349 | 33.9554 | Valerenic acid |
| M335T416 | 335.09471 | 416.128 | Nivalenol |
| M265T142 | 265.14345 | 141.5215 | (+)-abscisic acid |
| M135T260 | 135.11687 | 259.712 | Cedrol |
| M349T526 | 349.17177 | 526.447 | Diacetoxyscirpenol |
| M109T260 | 109.10112 | 259.964 | (cis+trans)-nerodilol |
| M555T256 | 555.24125 | 255.677 | Roridin a |
| M665T88 | 665.35018 | 87.5374 | Trichothecine |
| M175T255 | 175.14806 | 254.716 | alpha-ionone |
| M147T244 | 147.11679 | 243.931 | (-)-caryophyllene oxide |
| M201T252_3 | 201.16377 | 251.872 | .alpha.-cyperone |
| M203T259 | 203.17939 | 259.2765 | Kinoprene |
| M377T435 | 377.10548 | 435.244 | Fusarenone-x |
| M119T260 | 119.08549 | 259.699 | Zerumbone |
| M177T253 | 177.16372 | 252.527 | 7,8-dihydro-alpha-ionone |
| M237T251 | 237.18489 | 250.9835 | Curcumol |
| M565T215 | 565.18857 | 215.271 | Satratoxin f |
| M507T220 | 507.2201 | 220.3765 | Verrucarin j |

Table S12 Phenols in the acetone extract of *M. paniculata* leaf

| **ID** | **m/z** | **rt(s)** | **Name** | **SubClass** |
| --- | --- | --- | --- | --- |
| M207T134 | 207.06513 | 134.365 | Trans-3,5-dimethoxy-4-hydroxycinnamaldehyde | Methoxyphenols |
| M131T33 | 131.04855 | 33.058 | Coniferyl alcohol | Methoxyphenols |
| M209T275 | 209.08083 | 274.91 | Sinapyl alcohol | Methoxyphenols |
| M161T34 | 161.05954 | 34.32965 | Coniferyl aldehyde | Methoxyphenols |
| M183T97_1 | 183.06457 | 97.2821 | Syringaldehyde | Methoxyphenols |
| M151T27 | 151.03888 | 26.7214 | Vanillin | Methoxyphenols |
| M137T279 | 137.05967 | 279.132 | 4-hydroxy-3-methoxybenzyl alcohol | Methoxyphenols |
| M336T138 | 336.10531 | 137.704 | Feruloyl tyramine | Methoxyphenols |
| M134T457 | 134.04476 | 457.423 | Dl-normetanephrine | Methoxyphenols |
| M151T209 | 151.07532 | 209.405 | 3-methoxytyramine | Methoxyphenols |
| M155T180 | 155.08527 | 179.542 | 2,6-dimethoxyphenol | Methoxyphenols |
| M95T413 | 95.06046 | 413.084 | Phenol | 1-hydroxy-4-unsubstituted benzenoids |
| M107T213 | 107.04914 | 212.6 | 3-hydroxyphenylacetic acid | 1-hydroxy-4-unsubstituted benzenoids |
| M119T353 | 119.04921 | 353.17 | Dl-octopamine | 1-hydroxy-2-unsubstituted benzenoids |
| M150T43 | 150.05485 | 42.9846 | 4-hydroxymandelonitrile | 1-hydroxy-2-unsubstituted benzenoids |
| M152T261 | 152.07054 | 261.0935 | Acetaminophen | 1-hydroxy-2-unsubstituted benzenoids |
| M477T227 | 477.13673 | 226.921 | Methyl 3-(3,4-dihydroxyphenyl)-2-[[4-hydroxy-3-(3-methylbut-2-enyl)phenyl]methyl]-4-methoxy-5-oxofuran-2-carboxylate | Benzenediols |
| M109T313 | 109.02842 | 313.3475 | Pyrocatechol | Benzenediols |
| M194T356 | 194.11754 | 356.4405 | Isoproterenol | Benzenediols |
| M137T247 | 137.0597 | 246.7735 | Dobutamine | Benzenediols |
| M207T312 | 207.02879 | 311.812 | 3,4-dihydroxymandelic acid | Benzenediols |
| M109T389 | 109.02834 | 388.806 | 1,2,3-benzenetriol | Benzenetriols and derivatives |
| M153T213 | 153.05464 | 212.948 | 2-amino-4-nitrophenol | Nitrophenols |

Table S13 Other antibiotics in the acetone extract of *M. paniculata* leaf

| **ID** | **Name** | **KEGG** |
| --- | --- | --- |
| M693T287 | Monensin A | C06693 |
| M246T444_2 | Cycloheximide | C06685 |
| M539T61 | Fusidic acid | C06694 |
| M443T308 | Oligomycin b | C11312 |
| M798T46_1 | Oligomycin c | C11313 |
| M613T326 | Herbimycin a | C11225 |
| M266T469 | Anisomycin | C11281 |
| M544T446_3 | Adriamycin (Doxorubicin) | C01661 |
| M546T490 | Calcimycin | C11309 |
| M551T201_1 | Manumycin a | C12111 |
| M164T65 | 1-deoxynojirimycin | C16843 |
| M509T328 | Chrysomycin a | None |


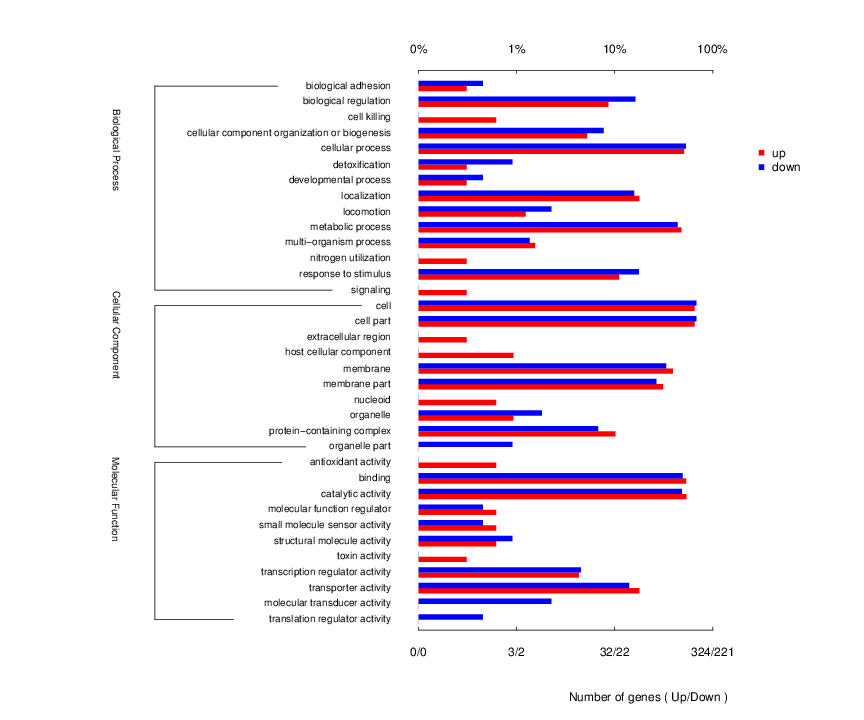
Figure S1 Barplot of *E. coli* differently expressed genes (mRNAs) based on GO enrichment analysis after treatment with acetone extract of *M. paniculata* leaf.


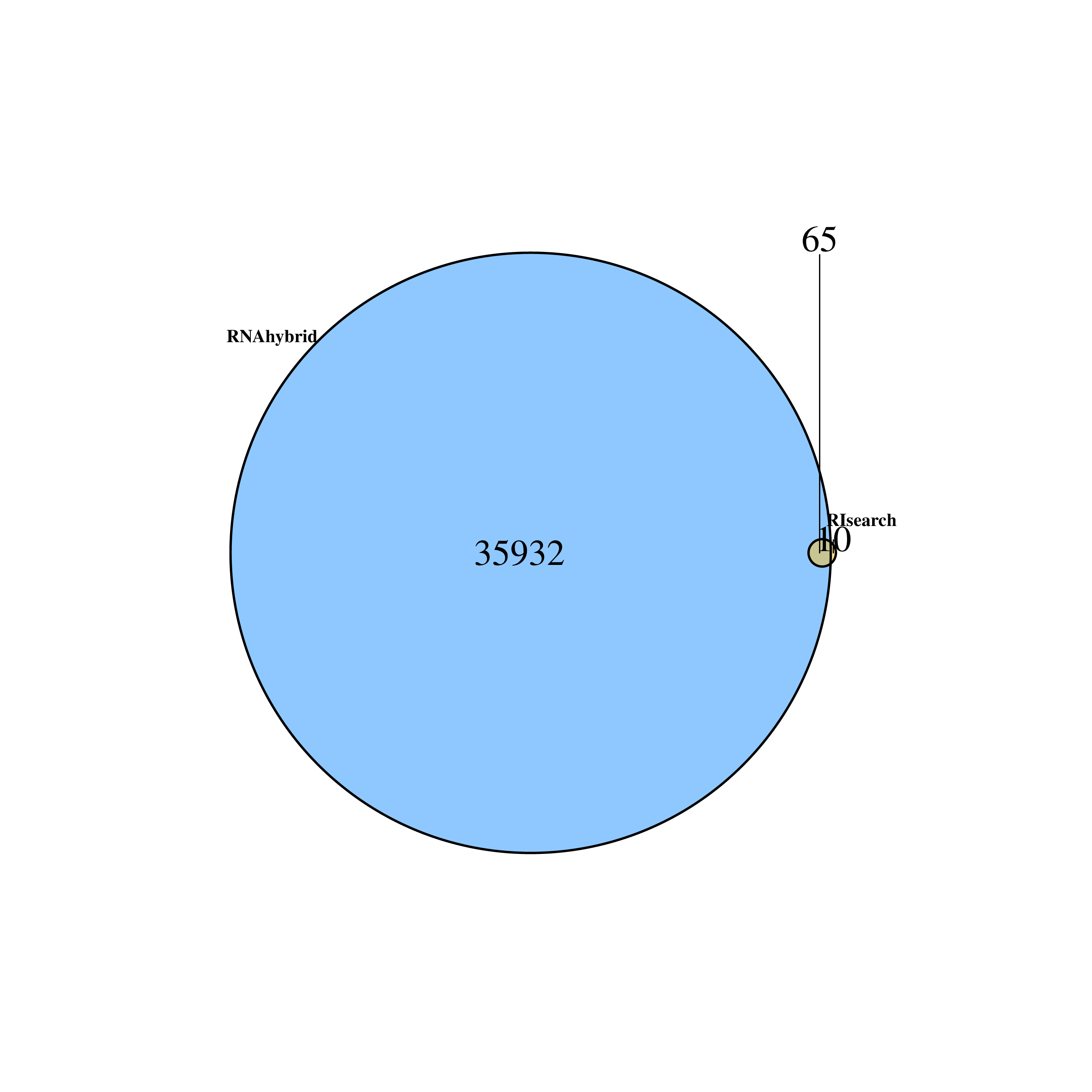


Figure S2 Venn diagram of sRNA target gene prediction by RNAhybrid and RIsearch softwires.


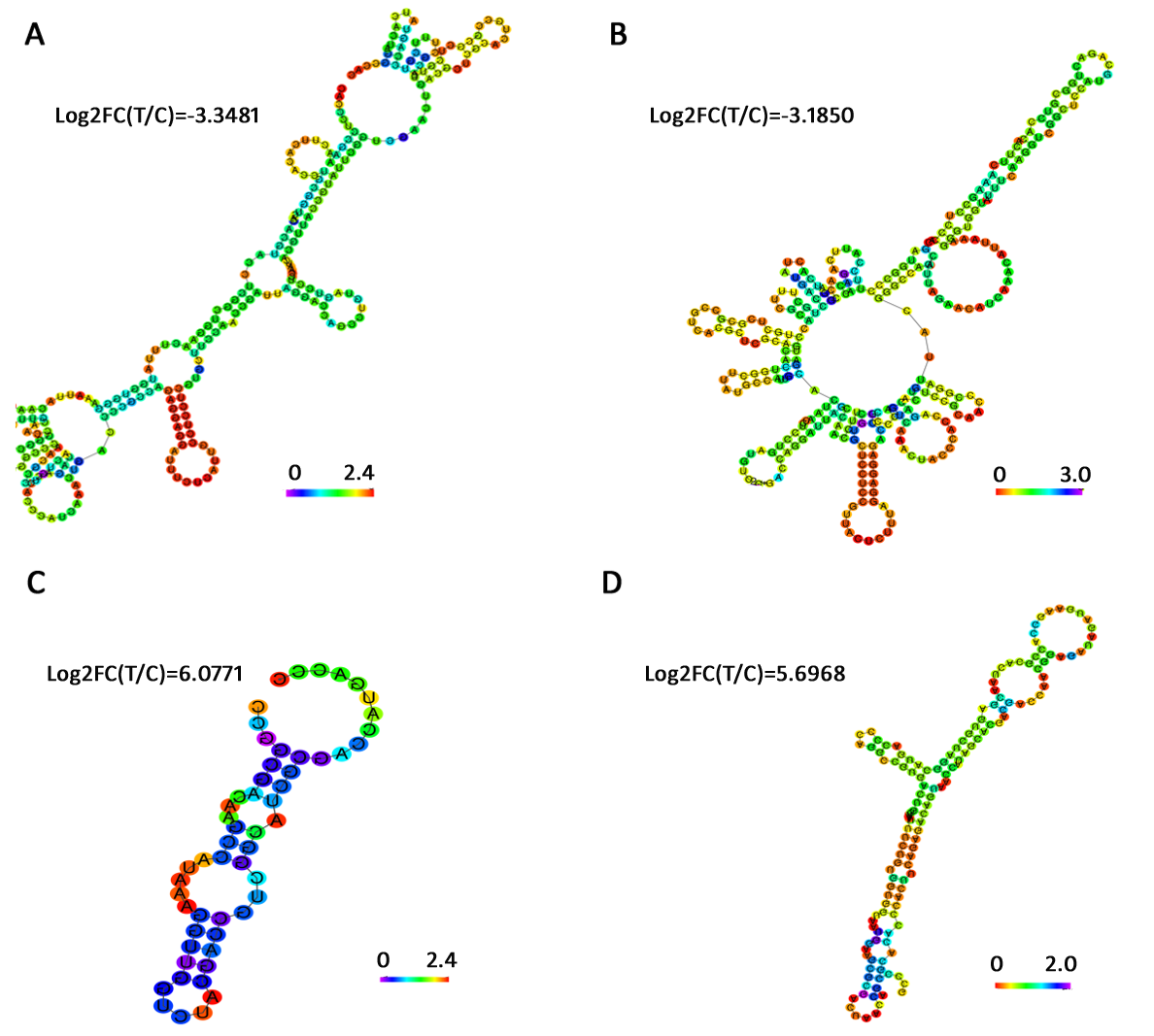


Figure S3 Structural illustration of the top regulated sRNAs in *E. coli* after treatment with AEML: A, the top upregulated sRNA (sRNA_298); B, the second upregulated sRNA (sRNA_57); C, the top downregulated sRNA (sRNA_335); D, the second downregulated sRNA (sRNA_336). C, control; T, treatment; AEML, acetone extract of *M. paniculata* leaf.


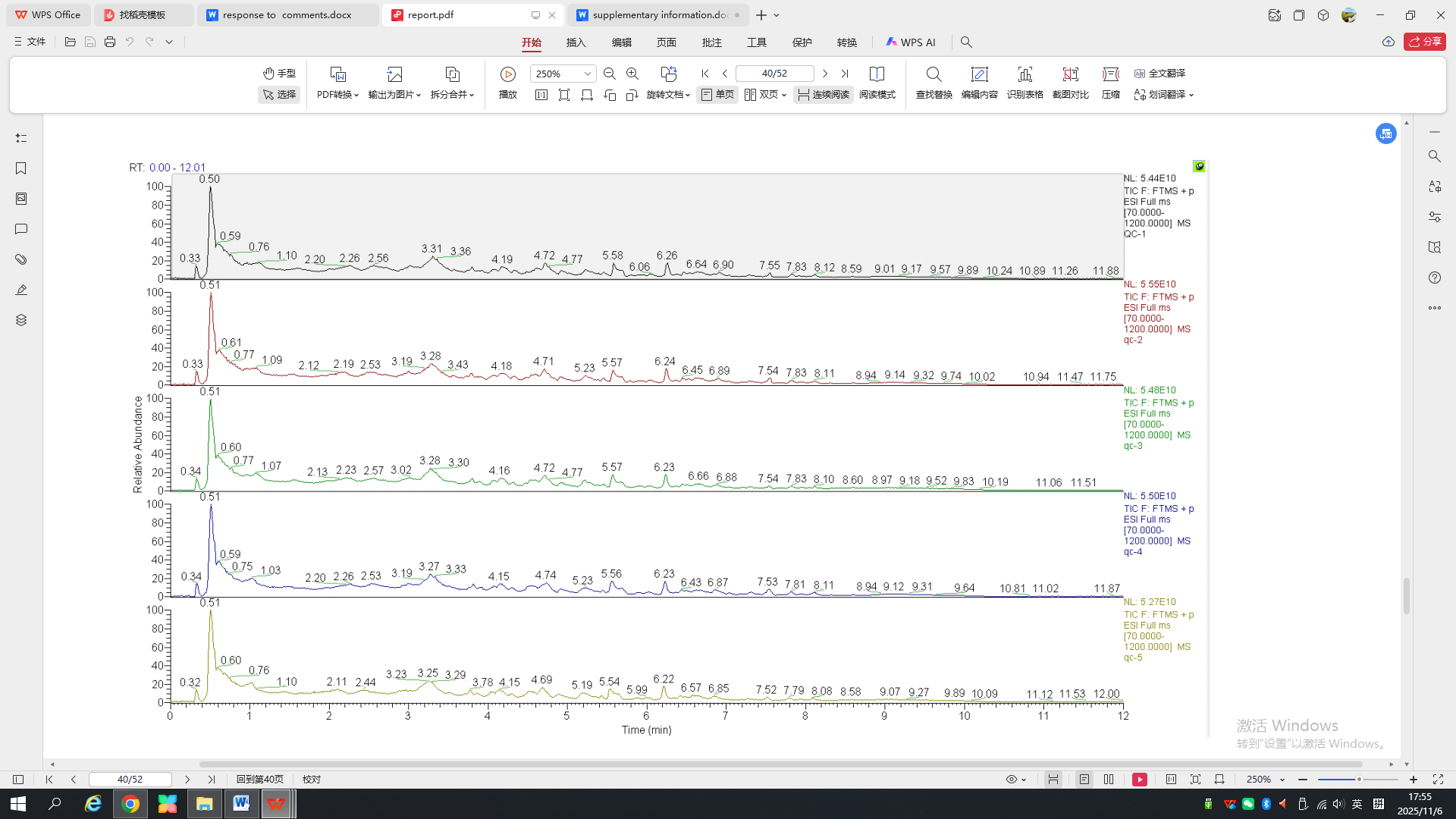


Figure S4 The total ion Chromatogram of QC under positive ion model


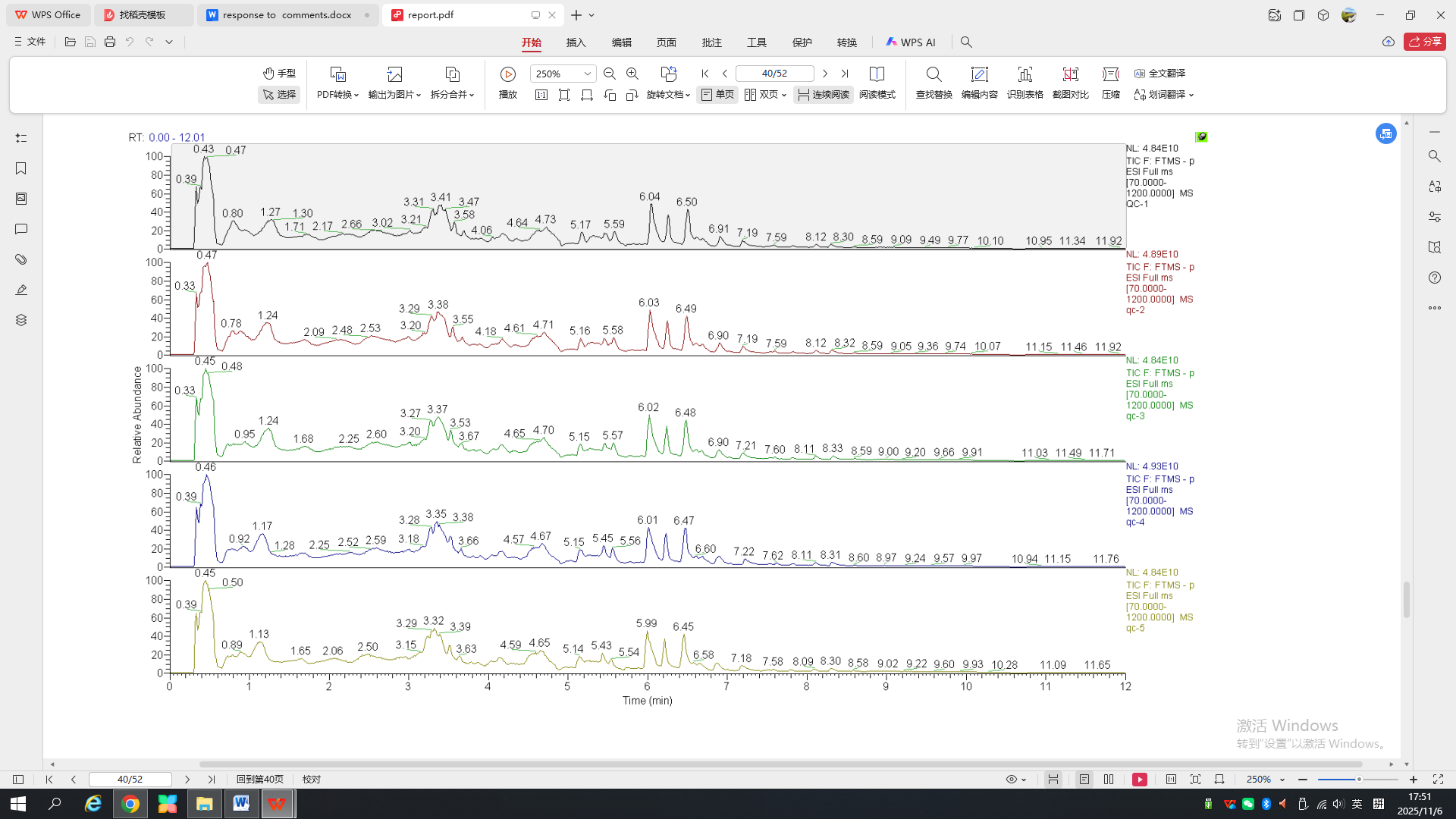


Figure S5 The total ion Chromatogram of QC under negative ion model

Figure S6 Detection of four antibiotics using ELISA in the acetone extract of *M. paniculata* leaf. Four antibiotics including Ampicilin (Amp), Norfloxacin (Nflx), Doxorubicin(Adr), and Tigecycline (Tgc), were detected by ELISA using corresponding commercial kits,
